# Advancements in MXene Composite Materials for Wearable Sensors: A Review

**DOI:** 10.3390/s24134092

**Published:** 2024-06-24

**Authors:** Bingqian Shao, Xiaotong Chen, Xingwei Chen, Shuzhe Peng, Mingxin Song

**Affiliations:** 1 School of Applied Science and Technology, Hainan University, Haikou 570228, China; 181233@hainanu.edu.cn (B.S.); 20213005493@hainanu.edu.cn (X.C.); 20213005415@hainanu.edu.cn (X.C.); 20213005551@hainanu.edu.cn (S.P.); 2 School of Electronic Science and Technology, Hainan University, Haikou 570228, China

**Keywords:** wearable electronics, MXene, sensors, nanocomposite

## Abstract

In recent years, advancements in the Internet of Things (IoT), manufacturing processes, and material synthesis technologies have positioned flexible sensors as critical components in wearable devices. These developments are propelling wearable technologies based on flexible sensors towards higher intelligence, convenience, superior performance, and biocompatibility. Recently, two-dimensional nanomaterials known as MXenes have garnered extensive attention due to their excellent mechanical properties, outstanding electrical conductivity, large specific surface area, and abundant surface functional groups. These notable attributes confer significant potential on MXenes for applications in strain sensing, pressure measurement, gas detection, etc. Furthermore, polymer substrates such as polydimethylsiloxane (PDMS), polyurethane (PU), and thermoplastic polyurethane (TPU) are extensively utilized as support materials for MXene and its composites due to their light weight, flexibility, and ease of processing, thereby enhancing the overall performance and wearability of the sensors. This paper reviews the latest advancements in MXene and its composites within the domains of strain sensors, pressure sensors, and gas sensors. We present numerous recent case studies of MXene composite material-based wearable sensors and discuss the optimization of materials and structures for MXene composite material-based wearable sensors, offering strategies and methods to enhance the development of MXene composite material-based wearable sensors. Finally, we summarize the current progress of MXene wearable sensors and project future trends and analyses.

## 1. Introduction

Since the debut of the inaugural wearable sensor in the 1960s [1], the realm of wearable technology has experienced extensive evolution over several decades. This progression has been catalyzed by advancements in IoT technologies, material science, and manufacturing methodologies. Presently, wearable sensors are advancing rapidly towards enhanced intelligence, reduced form factor, and greater integration. Within the diverse spectrum of wearable devices, flexible wearable electronic devices, underpinned by flexible sensors, stand out as a quintessential category [2]. These flexible sensors are broadly classified into various types, such as strain sensors [3,4], pressure sensors [5,6], and gas sensors [7,8]. The fundamental hardware architecture of flexible sensors uniformly encompasses a sensing module, an electrode module, and a substrate. The sensing module is tasked with data acquisition, the electrode module with signal transmission, and the substrate serves as the structural backbone. In the context of flexible wearable electronics, paramount factors include non-invasive detection capability and the comfort of the wearer. Here, the enhanced sensitivity of the sensing module, improved conductivity of the electrode module, and superior biocompatibility of the substrate emerge as pivotal elements in augmenting device performance. Consequently, the selection of materials that augment the sensing module’s sensitivity, boost the electrode module’s conductivity, and optimize the biocompatibility of the flexible substrate is a critical consideration in the advancement of sensor functionalities.

The progression in nanomaterial technology has heralded the widespread application of materials such as carbon nanotubes (CNTs) [9], silver nanowires (AgNWs) [10], cellulose nanofibers (CNFs) [11], and graphene [12] in the field of flexible electronics, chiefly attributed to their exceptional electrical conductivity and mechanical properties. Among these, two-dimensional (2D) materials have garnered considerable interest due to their distinctive mechanical, optical, thermal, and magnetic electronic characteristics [13]. Marking a significant milestone within the 2D materials category, 2D MXenes have been extensively employed across various domains including wearable devices [14], energy storage [15], catalysis [16], and electromagnetic interference shielding [17] since their initial synthesis in 2011. This synthesis, conducted by Naguib et al. [18], involved the etching of the Al layer from Ti_3_AlC_2_ at room temperature using hydrofluoric acid, leading to the creation of the 2D nanosheet Ti_3_AlC_2_. Predominantly, 2D MXenes are stratified transition metal carbides or nitrides. Their principal production process involves the selective etching of M_*n*+1_AX_*n*_(MAX) phase precursors with fluorine-based reagents. In this context, M stands for early transition metals, A denotes an element from group IIIA or IVA of the periodic table, X represents carbon or nitrogen, and the variable n ranges from 1 to 3 [19]. Studies have posited the theoretical feasibility of synthesizing over 100 variants of the MAX phase by varying the MAX combinations, thereby expanding the MXene production spectrum [20]. Despite the identification of more than 30 MXene materials in laboratory settings, research in the arena of flexible wearable sensors predominantly concentrates on the Ti_3_C_2_T_*x*_ form of MXene. Here, T_*x*_ symbolizes a variety of surface terminal groups (like O, OH, F, or Cl) acquired through distinct synthesis methodologies [21]. Thus, the performance of Ti_3_C_2_T_*x*_ can be effectively optimized by meticulous adjustment and characterization of its surface functional groups. In essence, Ti_3_C_2_T_*x*_ epitomizes a revolutionary 2D material, characterized by its high electrical conductivity, superior mechanical properties, and adaptable surface functionalization.

On the one hand, Ti_3_C_2_T_*x*_ materials, distinguished by their high electrical conductivity and superior mechanical properties, establish a solid material foundation for flexible sensors. This foundation underpins their exceptional sensing performance and notable extensibility [22]. On the other hand, the abundant functional groups present on the surface of Ti_3_C_2_T_*x*_ enable its effective integration with other nanomaterials. This synergy facilitates the creation of composite materials endowed with augmented functionalities [23]. Moreover, the comprehensive structural characterization of MXene materials permits the enhancement of their physicochemical attributes, which is achievable through the incorporation of microstructures and biomimetic strategies [24]. These remarkable properties endow MXene with great potential for applications in flexible wearable sensor fields such as strain sensing [25], pressure measurement [26], and gas detection [27].

In recent years, significant progress has been made in the research of MXene composites within the realm of wearable sensors [19,28,29]. While most review articles concentrate on the fundamental properties of MXene materials and their composites, along with their applications in wearable technology, there is a lack of detailed discussion regarding the specific sensing mechanisms and optimization strategies of these materials. For example, Ahmad et al. [19] examined the synthesis methods of MXene and the application of MXene-based gas sensors in environmental sensing. Grabowski et al. [28] reviewed the preparation and processing of MXene nanomaterials, their physical and chemical properties, and their application as sensing elements. Additionally, Ahmad’s group [29] summarized functional nanoarchitectures for MXene-based flexible electronics and system-level applications of MXene-based nanodevices. A crucial driving force behind the advancements in MXene composites for wearable sensor technology is the exploration of novel optimization strategies [30]. However, many related reviews have not thoroughly explored this aspect. To address this research gap, our study will focus on exploring and elaborating on optimization strategies for MXene composites, such as enhancing the flexibility and conductivity of these composites to improve their performance in wearable sensors.

**Figure 1:**
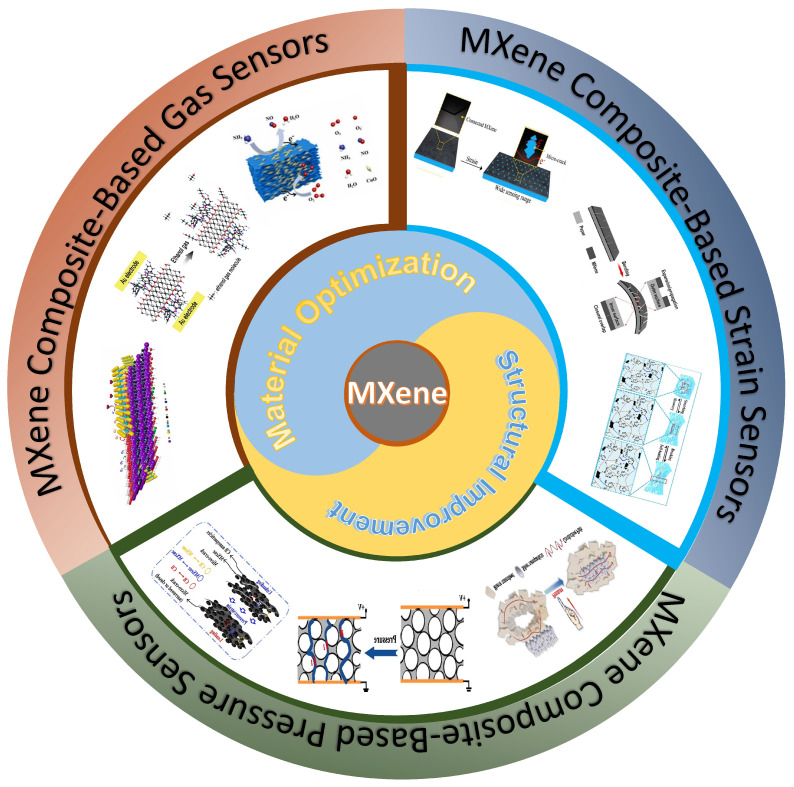
Overview of the latest developments in MXene composite material-based wearable sensors. Reprinted with permission from Refs. [31,32,33,34,35,36,37,38,39].

As illustrated in Figure 1, this paper reviews the recent advancements in strain sensors, pressure sensors, and gas sensors based on MXene composite materials. The review adopts a relatively parallel structure to sequentially introduce strain sensors, pressure sensors, and gas sensors based on MXene composite materials. While reviewing the aforementioned sensors, we emphasize the advantages of MXene composite materials over materials used in wearable sensor applications and elucidate the specific principles underlying these advantages in the field of wearable sensors. Additionally, we explore the specific principles behind the performance enhancement of wearable sensors based on MXene composite materials through the introduction of certain special structures. Finally, this review summarizes the overall situation of MXene composite materials in recent years and the challenges currently faced. Furthermore, based on the current status of wearable sensors utilizing MXene composite materials, we predict future research and development directions, as well as the potential challenges that may be encountered.

## 2. MXene Composite-Based Strain Sensors

Flexible strain sensors have been extensively applied in fields such as human–computer interaction, health management, and physiological activity monitoring due to their high sensitivity, simple structure, low cost, and portability, constituting a vital branch of wearable technology [14,40]. An ideal strain sensor should exhibit high sensitivity, excellent repeatability, and rapid response time, among other superior strain performance characteristics. Despite the widespread incorporation of conductive materials such as carbon-based materials, metals, and conductive polymers into the research of flexible sensors, limitations such as the high electrical resistance of carbon-based materials, the poor mechanical properties of metals, and the degradation of conductive polymers over prolonged use have hindered further advancements in the strain performance of flexible strain sensors [41]. Given the high electrical conductivity, outstanding mechanical properties, and corrosion resistance of MXene, the composite materials composed of the aforementioned material and MXene represent a feasible strategy for overcoming these limitations [42]. Herein, we summarize the latest developments in MXene-based composite material strain sensors over recent years, highlighting their potential to address the current challenges in the field (Table 1).

### 2.1. AgNWs-Based

**Figure 2:**
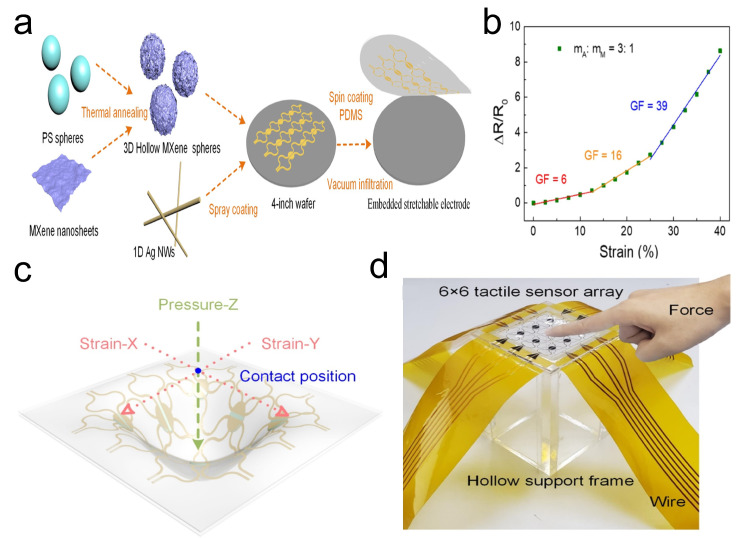
(**a**) Preparation of 3D hollow MXene sphere/AgNW multidimensional nanocomposite material and their structure. (**b**) Sensitivity of 3D hollow MXene sphere/AgNW multidimensional nanocomposite material. (**c**) Schematic diagram of the multiplexed detection principle. (**d**) Image of the multiplexed detection platform. Reprinted with permission from Ref. [43]. Copyright 2021, American Chemical Society.

AgNWs are a typical conductive material, distinguished by their excellent electrical conductivity, thermal conductivity, and mechanical flexibility, showing great potential in flexible strain sensors. However, flexible strain sensors utilizing single conductive nanomaterials often struggle to balance high conductivity with flexibility. Fortunately, combining them with suitable conductive nanomaterials presents an effective strategy to overcome this limitation [60]. Zhao et al. [43] designed a strain sensor using an embedded stretchable electrode based on a three-dimensional (3D) hollow MXene sphere/AgNW multidimensional nanocomposite material (Figure 2a). This multiscale hybrid conductor network, utilizing 3D hollow MXene spheres as local electron transport pathways and AgNWs as the main conductive routes, achieves a balance between conductivity and flexibility. Upon testing, the strain sensor exhibited a gauge factor (GF) of 39 within a strain range of 25–40%, with response/recovery times of 0.62/0.61 s, and demonstrated excellent repeatability over 2000 cycles at 20% strain (Figure 2b). Additionally, this strain sensor, in combination with a capacitive pressure sensor based on T-ZnOw/PDMS proposed by Zhao et al., forms a multiplexed detection platform capable of real-time monitoring of the magnitude, direction, and position of external forces (Figure 2c,d).

**Figure 3:**
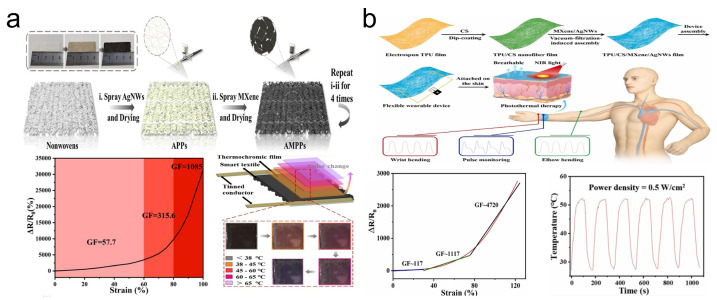
(**a**) Preparation of AMPP and its performance in strain sensing and temperature visualization. Reprinted with permission from Ref. [44]. Copyright 2021, American Chemical Society. (**b**) Preparation of the MXene/AgNWs/CS nano conductive fiber network and its performance in strain sensing (the red line represents the original data fitting curve, while the other three colored lines represent the segmented linear fitting curves of the original data) and thermal management. Reprinted with permission from Ref. [45]. Copyright 2023, Elsevier.

As the demand for wearable devices grows, there is an increasing need for flexible strain sensors with thermal management capabilities in addition to excellent strain sensing performance [61]. This requires the materials used in sensors to possess not only good electrical and mechanical properties but also exceptional thermal conductivity. Fortunately, research has shown that AgNWs and MXene materials meet these requirements [62,63]. Liu et al. [44] utilized AgNWs and MXene to construct a 3D conductive network and combined it with a PU-polypropylene nonwoven fabric (AMPP) to fabricate a flexible strain sensor with thermal management capability (Figure 3a). Due to the synergistic effect between AgNWs and MXene nanosheets within the 3D conductive network, the sensor achieved a high GF of 1085, was capable of sensing up to 100% strain, and had response/recovery times of 340/320 ms for 5% strain. In terms of thermal management, under simulated sunlight at 0.2 W/cm^2^, the AMPP rapidly reached a relative saturation temperature of 92.87 °C within continuous exposure for 120 s. Additionally, to prevent low-temperature burns, the sensor incorporated a temperature visualization feature. In another study, Chao et al. [45] designed a flexible, breathable, antibacterial strain sensor using a nano conductive fiber network composed of MXene, AgNWs, and breathable chitosan (CS) (Figure 3b). Tests showed that the sensor achieved a high GF of 4720, a low strain detection limit (0.0645%), and a wide sensing range (up to 120%). When exposed to near-infrared light at 0.5 W/cm^2^, the sensor reached 51 °C within 60 s, demonstrating excellent photothermal conversion performance.

**Figure 4:**
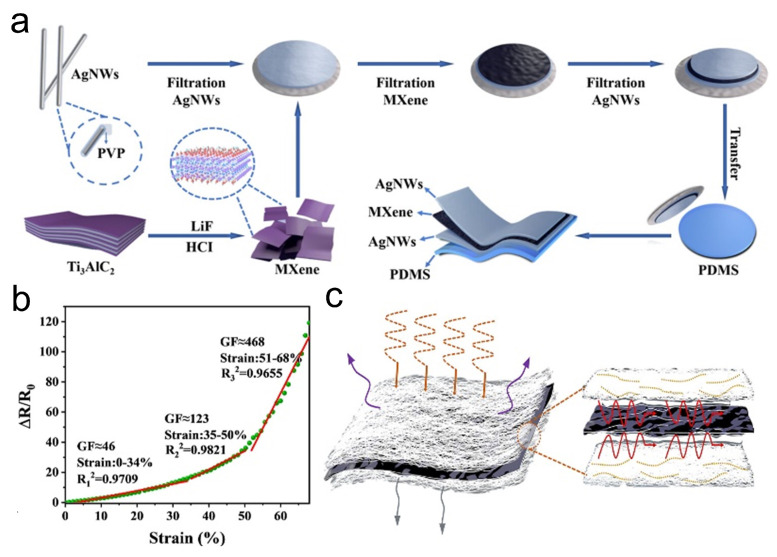
(**a**) Preparation of AgNW/MXene/PDMS composite films and their structure. (**b**) Performance of AgNW/MXene/PDMS in strain sensing, which the green dots represent the collected data points and the red line represents the fitted piecewise curve. (**c**) EMI shielding mechanism of AgNW/MXene/PDMS material. Reprinted with permission from Ref. [46]. Copyright 2023, American Chemical Society.

In everyday life, electromagnetic signals that are omnipresent can interfere with the signal transmission of wearable devices such as flexible strain sensors, making the development of electromagnetic interference (EMI) shielding materials crucial for stabilizing the performance of strain sensors [64]. The Bian group [46] constructed AgNW/MXene/ PDMS multifunctional composite films using a straightforward vacuum-assisted filtration process and transfer method (Figure 4a). The AgNWs effectively bridged the microcracks caused by strain between MXene sheets, enabling the AgNW/MXene/PDMS composite film to achieve a high GF of 468 and a wide sensing range (up to 68%)(Figure 4b). Additionally, as shown in Figure 4c, due to the MXene/AgNW layers’ ability to dissipate electromagnetic signals and the multiple internal reflections within the AgNW/MXene/PDMS composite film, it exhibited an excellent EMI shielding effectiveness of 50.82 dB.

### 2.2. CNTs-Based

**Figure 5:**
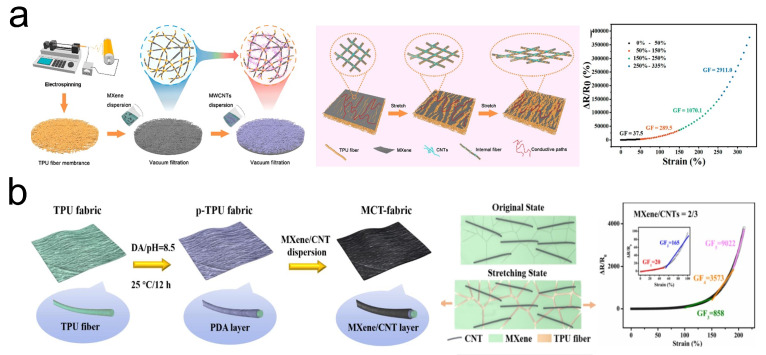
(**a**) Preparation of MXene/CNT/TPU composite film and its sensing mechanism and performance in strain sensing. Reprinted with permission from Ref. [47]. Copyright 2022, American Chemical Society. (**b**) Preparation of MCT-fabric and its sensing mechanism and performance in strain sensing. Reprinted with permission from Ref. [48]. Copyright 2022, Elsevier.

CNTs, as a significant member of the carbon-based material family, are deemed to have vast potential in flexible strain sensors due to their excellent mechanical properties, high electrical conductivity, superior chemical durability, and good biocompatibility [65]. However, like other nanomaterials, individual CNT materials often undergo self-stacking, which can impair the strain performance of CNT-based strain sensors. A solution to this issue involves the incorporation of MXene conductive materials into the CNT dispersion [66]. Dong et al. [47] reported the development of a typical bilayer conductive structure Ti_3_C_2_T_*x*_ MXene/CNT/TPU composite film, using a porous electrospun TPU mat as the scaffold, through a simple and scalable vacuum filtration process (Figure 5a). Thanks to the bilayer structure, the strain sensor based on Ti_3_C_2_T_*x*_ MXene/CNT/TPU composite film achieved a high GF (GF = 2911), a wide sensing range (up to 330%), and outstanding long-term durability (2600 cycles at 50% strain). Additionally, this sensor was capable of detecting subtle strains as small as 0.1%. In another work, Zhang et al. [48] fabricated multifunctional electronic textiles (MCT-fabric) by a simple dip-coating method using a blend of Ti_3_C_2_T_*x*_ MXene and CNT conductive nanomaterials coated on TPU nonwoven fabric (Figure 5b). To further enhance the sensitivity of MCT-fabric, Zhang et al. also created ultra-sensitive microcrack structures through pre-stretching. Tests showed that the MCT-fabric had a GF as high as 9022, a sensing range up to 210%, long-term durability (1000 cycles at 50% strain), and a detection limit for subtle strains of 0.1%. Moreover, in terms of thermal management, the MCT-fabric could reach a specified temperature within 4 s under a voltage of 5 V and maintain stability for 1000 s, demonstrating excellent thermal management capability.

**Figure 6:**
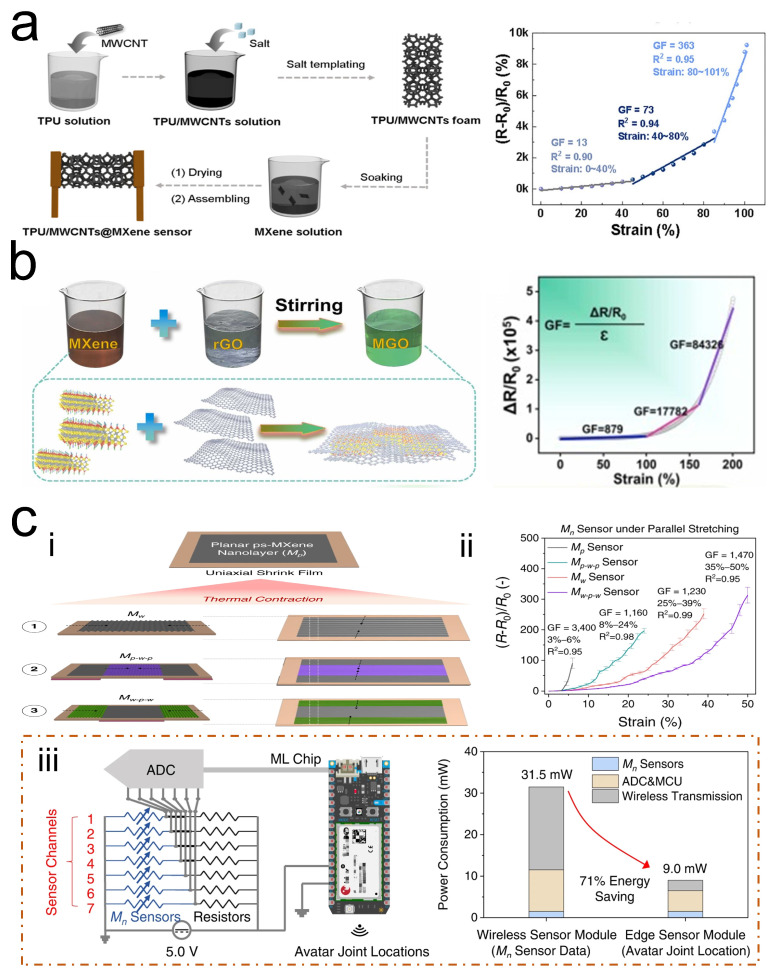
(**a**) Preparation of TPU/MWCNTs @MXene foam sensor and its performance in strain sensing. Reprinted with permission from Ref. [49]. Copyright 2021, American Chemical Society. (**b**) Preparation of MGTSS and its performance in strain sensing. Reprinted with permission from Ref. [50]. Copyright 2021, Elsevier. (**c**) SWCNT/MXene/PVA strain sensor: (**i**) preparation of SWCNT/MXene/PVA strain sensor; (**ii**) performance of four SWCNT/MXene/PVA strain sensors in strain sensing; (**iii**) application of SWCNT/MXene/PVA strain sensor in the sensor modules. Reprinted with permission from Ref. [51]. Copyright 2022, Nature.

Research has demonstrated that strain sensors with foam-like 3D structures not only possess the capability to detect various types of strain but also offer advantages such as breathability and light weight [67]. Inspired by this, Wang et al. [49] utilized a combination of the salt templating method and dip-coating technique to fabricate a TPU/MWCNT @MXene foam sensor composed of TPU, MWCNT, and Ti_3_C_2_T_*x*_ MXene (Figure 6a). Owing to its 3D foam-like structure, this sensor achieved a high GF of 363 and a wide detection range (up to 101%). Moreover, constructing biomimetic structures is a common strategy used to enhance the sensing performance of strain sensors. For instance, inspired by the structure of nacre, Xu et al. [50] reported a strain sensor based on an MXene/rGO/TPU strain sensor (MGTSS) (Figure 6b).Thanks to the nacre-mimetic structure and the synergistic movement of MXene/rGO nanosheets, the MGTSS not only exhibited an extremely high GF (GF = 84,326) but also provided a wide sensing range (up to 200%) and a detection limit for strain as low as 0.05%.

With the advancement of science and technology, optimization of strain sensors can be achieved not only through the improvement of materials and structures but also by leveraging integrated chip technology. Yang et al. [51] developed four types of strain sensors with strain detection capabilities based on three building materials: SWCNT, Ti_3_C_2_T_*x*_ MXene nanosheets, and PVA (Figure 6c). By employing a method that creates wrinkle-like morphologies through localized thermal shrinkage, the four sensors achieved a maximum detection range of 84%, with GF exceeding 1000 for all sensors. Furthermore, utilizing these sensors integrated with a machine learning chip resulted in sensor modules capable of reconstructing personalized avatar animations to mimic various full-body movements without the need for external computing devices, with an average avatar positioning error of 3.5 cm. Additionally, compared to wireless sensor modules, the power consumption for avatar reconstruction in Yang et al.’s standalone sensor module was reduced by 71%, providing an excellent example of the benefits of integrating chips into strain sensors.

### 2.3. CNFs-Based

**Figure 7:**
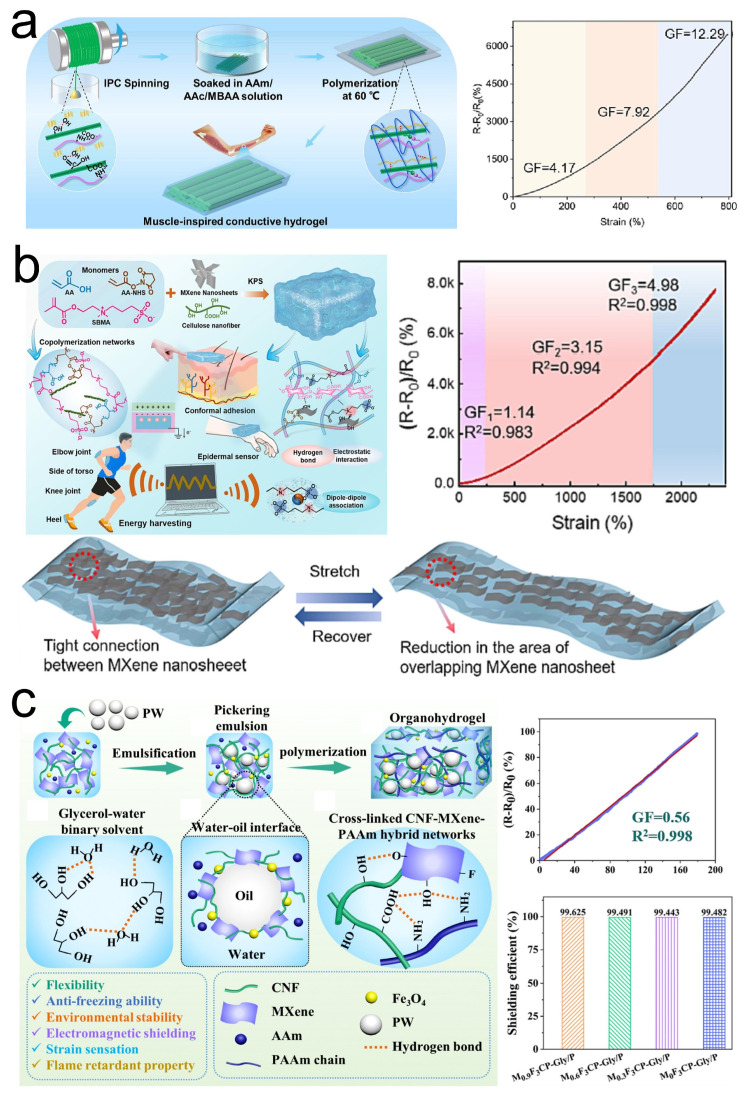
(**a**) Preparation of the CNF/MXene dual-network hydrogel and its performance in strain sensing. Reprinted with permission from Ref. [52]. Copyright 2023, Elsevier. (**b**) Preparation of PAS/MXene/CNF hydrogels and their performance and schematic mechanism in strain sensing. Reprinted with permission from Ref. [53]. Copyright 2023, Elsevier. (**c**) Preparation of MFCP-Gly/P organohydrogels and their performance in strain sensing and EMI. Reprinted with permission from Ref. [54]. Copyright 2022, Elsevier.

CNF is renowned for its excellent flexibility, ease of manufacturing, and good electrical conductivity, offering significant promise for low-cost and large-scale industrial production of flexible strain sensors. Inspired by the oriented fibrous layered structure of muscle tissue, Geng et al. [52] designed an anisotropic CNF/MXene dual-network hydrogel (Figure 7a). This hydrogel, characterized by a directional, rigid, and densely crosslinked first network and an isotropic, flexible, and loosely crosslinked second network, possesses excellent mechanical properties and electrical conductivity. As tested by Geng et al., the sensing range can reach up to 800%, with a maximum gauge factor (GF) of 12.29. In another study, Zhang et al. [53] utilized MXene and CNF as crosslinkers and nanoscale conductive fillers to polymerize AA, AA-NHS, and SMBA into PAS/MXene/CNF hydrogels (Figure 7b). The PAS/MXene/CNF hydrogel exhibits favorable sensing performance within a strain range of 0-2240%, with a maximum GF of 4.98. Moreover, the PAS/MXene/CNF hydrogel demonstrates good adhesion, attributed to various non-covalent interactions within the PAS/MXene/CNF network and covalent interactions between NHS esters at the PAS/CNF/MXene hydrogel–tissue interface and amino groups in tissues. Additionally, He et al. [54] fabricated a flexible organic hydrogel (MFCP-Gly/P organohydrogels) filled with MXene/Fe_3_O_4_ shell-encapsulated paraffin microspheres based on stable cellulose Pickering emulsions (Figure 7c). Upon testing, the MFCP-Gly/P organohydrogels exhibited a GF of 0.56 within a strain range of 0–181.5%. Furthermore, the conductive pathways formed by MXene and the scattering attenuation effect of the MXene/Fe_3_O_4_ shell structure on electromagnetic waves enable the MFCP-Gly/P organohydrogels to effectively shield 99.625% of electromagnetic waves.

### 2.4. Conductive Hydrogels-Based

**Figure 8:**
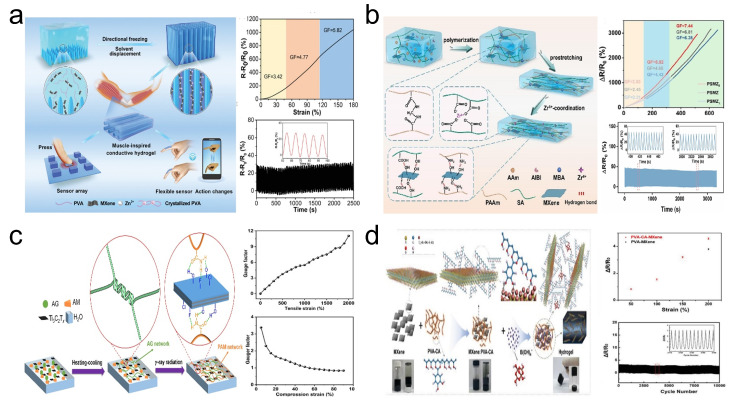
(**a**) Preparation of PMZn-GL hydrogel and its performance in strain sensing. Reprinted with permission from Ref. [55]. Copyright 2021, Wiley Online Library. (**b**) Preparation of PSMZ hydrogel and its performance in strain sensing. Reprinted with permission from Ref. [56]. Copyright 2023, Elsevier. (**c**) Preparation of AG/T-PAM hydrogel and its performance in strain sensing. Reprinted with permission from Ref. [57]. Copyright 2022, Elsevier. (**d**) Preparation of PVA-CA-MXene hydrogel and its performance in strain sensing. Reprinted with permission from Ref. [58]. Copyright 2023, Wiley Online Library.

Due to their good electrical conductivity, excellent mechanical properties, high biocompatibility, and self-healing characteristics, conductive hydrogels have garnered significant interest from researchers in the field of flexible and wearable device applications [68]. Structurally, hydrogels are soft solids composed of a 3D hydrophilic polymer network with a high water content. Interestingly, biological tissues such as muscles and skin can essentially be considered as hydrogels composed of biomolecules and possessing anisotropic characteristics. Research has shown that anisotropic structures can significantly improve the material’s electrical conductivity. Inspired by muscle structure, Feng et al. [55] created an anisotropic PMZn-GL hydrogel by mixing PVA, MXene nanosheets, and ZnSO_4_ solution, followed by directional freezing and binary solution replacement methods (Figure 8a). The anisotropic and ordered structure of the PMZn-GL hydrogel enhanced its mechanical and electrical properties in a specific direction, achieving a GF of about 3.42 within a strain range of 0–50% and 250 cycles at 30% strain. In another study, Guo et al. [56] reported an anisotropic hydrogel (PSMZ) obtained by coordinating zirconium (Zr^4+^) ions with a polyacrylamide-sodium alginate-MXene (PSM) hydrogel that features a dual-network (DN) structure (Figure 8b). Tests showed that PSMZ could achieve a GF of 2.83 within a 0–120% strain range and a high strain capacity of up to 570%.

**Figure 9:**
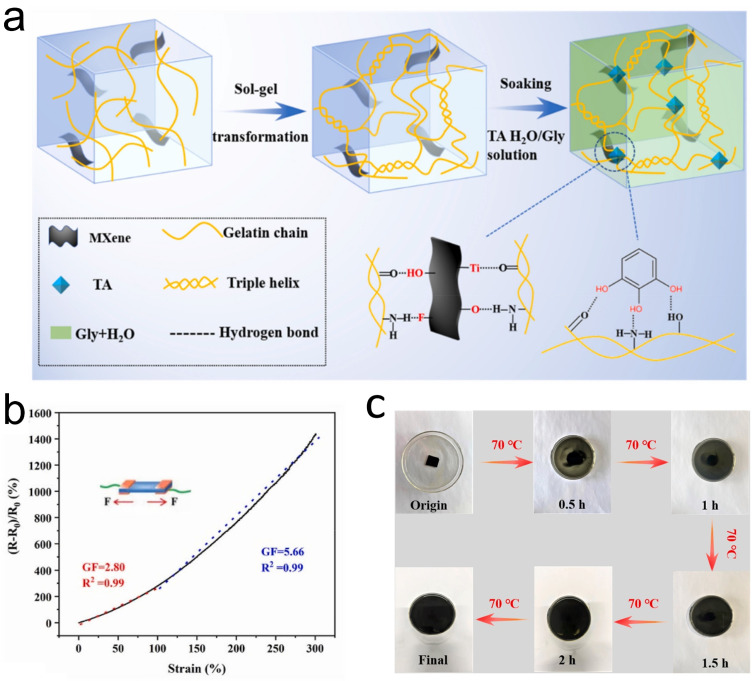
(**a**) Preparation of MCG organic hydrogel. (**b**) Performance of MCG organic hydrogel in strain sensing. (**c**) Decomposition process of MCG organic hydrogel placed in water. Reprinted with permission from Ref. [59]. Copyright 2022, Elsevier.

As previously mentioned, hydrogels are characterized by their high water content. However, water molecules and dissolved oxygen in water can lead to the oxidation of MXene, resulting in a decrease in the electrical conductivity of MXene-containing hydrogels [69]. Overcoming the oxidation of MXene is a crucial issue that needs to be addressed for the further development of MXene-containing hydrogels. Lin et al. [57] synthesized an agarose/Ti_3_C_2_T_*x*_ (AG/T-PAM) DN hydrogel through a method combining heating–cooling and γ-ray radiation-induced polymerization (Figure 8c). The polymer network within the AG/T-PAM hydrogel can prevent oxygen in the air from entering the hydrogel, ensuring a shelf life of up to one year with proper sealing. The hydrogel achieved a GF of 3.38 under 5% compressive strain and a GF of 11.1 under 2000% strain. Due to the characteristic of catechol to form tight bonds with the surface of MXene, catechol holds significant potential in inhibiting the oxidation of MXene [70]. For example, the team led by Chae [58] proposed a method to fabricate PVA-CA-MXene hydrogels by utilizing a polyvinyl alcohol–carboxylic acid (PVA-CA) polymer functionalized with catechol, obtained through a one-step reaction between PVA and 3,4-dihydroxybenzaldehyde, for the treatment of MXene (Figure 8d). The presence of catechol, which can form tight bonds with the MXene surface, effectively inhibits the oxidation of MXene. Meanwhile, the MXene-PVA-C hydrogel could achieve a GF of 2.3 under 200% strain and exhibit ultra-high repeatability (10,000 cycles at 150% strain). In another work, Wang et al. [59] reported an MXene composite gelatin (MCG) organic hydrogel based on MXene and gelatin (Figure 9a). Gelatin contains rich groups, including catechol functional groups, which can effectively adsorb onto MXene materials and thus prevent MXene oxidation. On one hand, the MCG organic hydrogel exhibited good strain performance with a GF of 2.80 within a 100% strain range (Figure 9b). On the other hand, the MCG organic hydrogel not only possesses good degradability (as shown, it can completely degrade in hot water at 70 °C within 2 h) but its degradation products are also non-toxic and harmless, showing the potential application in the field of green flexible strain sensors (Figure 9c).

## 3. MXene Composite-Based Pressure Sensors

To date, researchers have devoted considerable effort to the study of pressure sensors. From the perspective of sensing mechanisms, the mechanisms of reported flexible pressure sensors can be categorized into capacitive [71,72], piezoelectric [73,74], triboelectric [75,76], and piezoresistive effects [77,78]. Compared with other types of pressure sensors, piezoresistive sensors, composed of flexible substrates and conductive materials, are considered ideal for the next generation of pressure sensors due to their high sensitivity, fast response, good stability, and low manufacturing cost [79,80]. For piezoresistive flexible pressure sensors, sensitivity and sensing range are crucial metrics for evaluating sensor performance. Research indicates that the conductive performance and structure of the sensing materials significantly influence the sensor’s sensitivity [81]. Therefore, the choice of sensing materials greatly affects the sensor’s sensing performance. MXene, as an emerging class of 2D transition metal carbides and nitrides, is considered to have tremendous potential for the sensitive layers of pressure sensors due to its excellent electrical conductivity and interesting layered structure [82]. Additionally, MXene’s exceptional hydrophilicity makes it suitable for common flexible device fabrication processes, including filtration, spraying, dip-coating, screen printing, and inkjet printing [83,84,85]. In recent years, flexible pressure sensors based on MXene have been extensively studied. In this section, we summarize the development of MXene pressure sensors over the past few years from the perspective of material classification (Table 2).

### 3.1. AgNWs-Based

**Figure 10:**
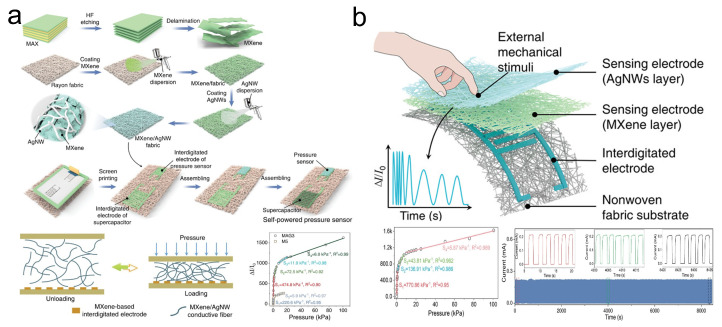
(**a**) Preparation of MXene/AgNWs-decorated nonwoven fabric and its performance and schematic mechanism in pressure sensing. Reprinted with permission from Ref. [86]. Copyright 2023, Wiley Online Library. (**b**) Schematic structure of MAF-based pressure sensor and its performance in pressure sensing. Reprinted with permission from Ref. [87]. Copyright 2023, Wiley Online Library.

In electronic textiles’ piezoresistive sensors, the integration of micro/nanostructures significantly enhances the sensitivity of piezoresistive sensors [98]. Research indicates that the composite structure formed by AgNW and MXene nanosheets features abundant surface micro/nanostructures [99]. Furthermore, blending MXene nanosheets with AgNWs can improve the electrical conductivity of MXene [100]. Therefore, the incorporation of MXene/AgNW composite materials is an effective strategy to boost the sensitivity of piezoresistive sensors. For instance, Zheng et al. [86] introduced a multifunctional MXene/AgNW3-decorated textile (MAG3) (Figure 10a). The substantial contact area provided by MAG3, coupled with its superior conductivity, plays a pivotal role in enhancing sensitivity. The pressure sensor based on MAG3 achieves a peak sensitivity of 474.8/kPa within a sensing range of 0-100 kPa. This sensor has a minimum detectable limit of 1 Pa and exhibits a response/recovery time of 140/30 ms at 0.5 kPa pressure. Additionally, breathability is one of the essential aspects of wearable pressure sensors, influencing their comfort in use. To enhance sensor breathability, Zheng et al. [87] utilized an expandable screen printing technique to fabricate a flexible pressure sensor with outstanding breathability and sensing performance based on MXene/AgNW materials (Figure 10b). By combining MXene/AgNW sensing electrodes with MXene-based interdigital electrodes, they developed a flexible MXene/AgNW fabric (MAF) pressure sensor. Owing to its multilayered and porous structure, the MAF pressure sensor exhibits high breathability (607 mm/s). The multi-layered and porous configuration of the MAF pressure sensor not only contributes to exceptional breathability but also significantly enhances sensitivity. The rough and porous surface of the nonwoven fabric allows for the formation of more conductive networks under external pressure by the MXene-based interdigital electrodes. As a result, the MAF pressure sensor achieves high sensitivity (770.86/kPa) and a broad sensing range (0–100 kPa). Even after 5000 cycles of compression testing, the sensitivity retention of MAF only decreased by 2.7%. Moreover, this sensor is capable of achieving a response/recovery time of 71/80 ms at 1.25 kPa and can also detect slight pressures of 1 Pa, showcasing its potential in low-pressure detection applications.

### 3.2. Carbon Nanomaterials-Based

**Figure 11:**
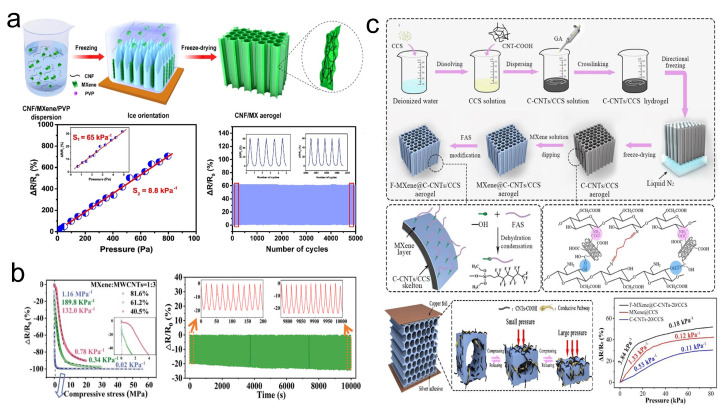
(**a**) Preparation of carbon nanofiber/MX with ordered microchannel architecture aerogel and its performance in pressure sensing. Reprinted with permission from Ref. [88]. Copyright 2021, Elsevier. (**b**) Performance of TPU/MXene/MWCNTs composite pressure sensor with TPMS structure in pressure sensing. Reprinted with permission from Ref. [89]. Copyright 2023, Wiley Online Library. (**c**) Preparation of F-MXene@C-CNTs/CCS aerogel and its schematic mechanism and performance in pressure sensing. Reprinted with permission from Ref. [90]. Copyright 2021, Elsevier.

Research has demonstrated that conductive networks constructed from single sensing materials inevitably face limitations due to their structural characteristics, particularly the unavoidable agglomeration and stacking of 2D nanosheets within polymer matrices. This challenge makes it exceedingly difficult to balance a wide detection range with high sensitivity in sensors. To address this issue, hybridizing materials of different dimensions have been proposed [101]. Conductive polymer composites, composed of polymers and conductive nanofillers, exhibit high conductivity and excellent flexibility [102]. Thus, combining MXene with conductive carbon nanomaterials is a promising approach to fully leverage the advantages of MXene in flexible devices. The Qin group [88] fabricated a carbon nanofiber/MXene (carbon nanofiber/MX-s) with ordered microchannel architecture aerogel using a unidirectional freeze-casting and lyophilization process assisted by liquid nitrogen, where “s” represents the mass ratio of carbon nanofiber to MXene (Figure 11a). This ordered microchannel architecture in the sensor leads to a decrease in the resistance of carbon nanofiber/MX-1 under compression, thereby affecting the sensitivity of the carbon nanofiber/MX-1 piezoresistive sensor. The sensitivity of carbon nanofiber/MX-1 remained stable after 5000 cycles of 50% strain compression, with a detection limit as low as 5 Pa and a sensitivity of up to 65/kPa for external pressures below 8 Pa. Li et al. [89] introduced a flexible pressure sensor based on a triply periodic minimal surface (TPMS) and hybrid MXene/MWCNTs material (Figure 11b). By embedding the hybrid MXene/MWCNTs filler into porous TPU and integrating it with a TPMS structure, the resultant porous TPU/MXene/MWCNTs (TMM) composite sensor could achieve continuous variation in the contact area under external pressure, endowing the TMM pressure sensor with outstanding piezoresistive sensing characteristics. With a porosity of 40.5%, the TMM pressure sensor exhibited higher sensitivity (132/kPa) and a much wider pressure range (0–5.7 MPa) compared to some recently reported sensors with high sensitivity or broad response ranges [103,104,105]. During a 10% strain compression cycle at a speed of 8 mm/min over 10,000 s, the TMM pressure sensor showed very stable resistance changes within 0–200 s and 9800–10,000 s. Beyond enhancing sensing performance, considerations like the hydrophobicity of sensors should also be taken into account for everyday applications. Yang et al. [90] fabricated a carboxylated carbon nanotubes/carboxymethyl chitosan ((F-MXene)@C-CNTs/CCS) aerogel with a honeycomb-like porous microstructure, surface-modified by FAS-treated MXene through a simple directional freeze–drying method (Figure 11c). The crosslinked network formed by C-CNTs and CCS using glutaraldehyde as the crosslinker, along with the honeycomb-like microstructure, not only endowed the (F-MXene)@C-CNTs/CCS with excellent mechanical properties but also maintained stability in sensitivity after 1000 compression cycles at 30% strain. Furthermore, thanks to the honeycomb-like microstructure and conductive pathways provided by MXene under compressive stress, the (F-MXene)@C-CNTs/CCS achieved a sensitivity of up to 3.84/kPa within a sensing range of 0 to 80.9 kPa. Additionally, the hydrophobicity afforded by FAS modification allowed the (F-MXene)@C-CNTs/CCS to maintain stable sensing performance even in the presence of water and sweat over 200 test cycles.

### 3.3. CNFs-Based

**Figure 12:**
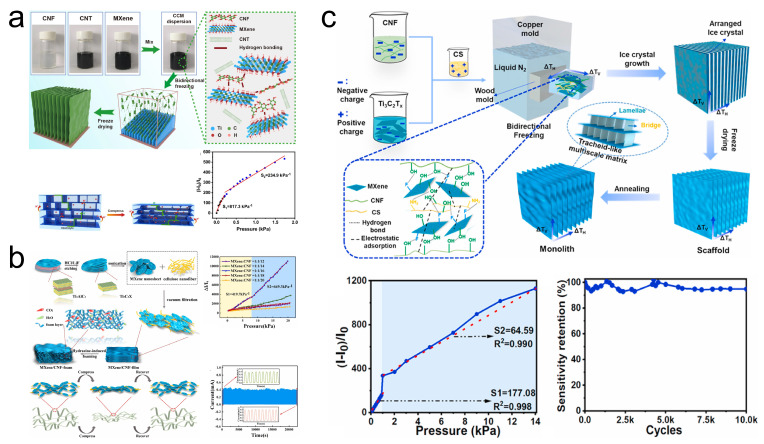
(**a**) Preparation of CNF/CNT/MXene aerosol and its schematic mechanism and performance in pressure sensing. Reprinted with permission from Ref. [91]. Copyright 2023, Springer. (**b**) Preparation of MXene/CNF-foam and its schematic mechanism and performance in pressure sensing. Reprinted with permission from Ref. [92]. Copyright 2021, Elsevier. (**c**) Preparation of MXene/CNF/CS pressure sensor and its schematic mechanism and performance in pressure sensing. Reprinted with permission from Ref. [93]. Copyright 2023, Elsevier.

Research has indicated that the interlayer spacing of MXene films, utilized in pressure sensors, diminishes due to the aggregation and self-stacking induced by van der Waals forces and hydrogen bonds between adjacent MXene nanosheets, consequently impeding the sensors’ sensitivity performance [106]. Introducing intercalating agents to bond with MXene nanosheets, thereby expanding their interlayer spacing, presents a viable solution to this challenge. The incorporation of one-dimensional (1D) CNF is noteworthy, as their high aspect ratio and 1D nanofiber structure ensure maintained overall conductivity post-integration with MXene. Furthermore, CNFs are advantageous due to their low cost, lightweight, renewability, and environmental friendliness [107,108], making them an attractive intercalating material to enhance the interlayer spacing of MXene nanosheets. Xu et al. [91] introduced a piezoresistive flexible pressure sensor fabricated using CNF/CNT/MXene aerogel prepared via a simple bidirectional freezing method (Figure 12a). The sensor benefits from the enhanced roughness and tubular structure provided by nano CNFs and CNTs wrapped around the CNF/CNT/MXene aerogel surface, forming multiple conductive pathways internally when subjected to external pressure. Consequently, this sensor achieves sensitivities of 817.3/kPa in the 0–200 Pa range and 234.9/kPa in the 200–1500 Pa range. Additionally, the pressure sensor based on CNF/CNT/MXene aerogel maintains stable sensing performance with excellent response/recovery times (74/50 ms) after 2000 compression loading/unloading cycles at 30% strain. Su et al. [92] utilized vacuum filtration and hydrazine hydrate-induced foaming processes to fabricate MXene/CNF composite foam, assembling it into a piezoresistive pressure sensor with a PI flexible substrate, copper wire electrodes, and a polypropylene film, named MXene/CNF-foam sensor (Figure 12b). Due to the face-to-face contact across different dimensions of the MXene/CNF composite foam, the sensor exhibits high sensitivity and a wide sensing range: achieving a sensitivity of 419.7/kPa within 0–8.04 kPa and up to 649.3/kPa within the 8.04–20.55 kPa higher pressure range. The porous layered structure endows the MXene/CNF composite foam with exceptional mechanical properties, maintaining sensitivity stability and showcasing response/recovery times of 123/139 ms after 10,000 compression loading/unloading cycles. Intriguingly, the MXene/CNF-foam can be completely degraded by a low concentration of H_2_O_2_ solution, aligning well with green and sustainable development objectives. Not only can the introduction of CNF overcome the challenges of MXene for pressure sensors, but the incorporation of MXene can also address some of the shortcomings of CNF-related materials for pressure sensors. Wei et al. [93] devised a carbon-based aerogel with a tubular structure composed of MXene, CNF, and CS. The introduction of MXene and CS materials, along with the design of the tubular structure, mitigated the mechanical performance deficiencies observed in CNF-based carbon aerogels. Upon evaluation, the pressure sensor derived from this aerogel exhibited sensitivities of 117.08/kPa and 64.59/kPa within the ranges of 0–1 kPa and 1–12 kPa, respectively, with a minimum detectable force of 3 Pa. Furthermore, even under a 50% compressive strain and after 10,000 cycles, the pressure sensor maintained a stable response.

### 3.4. Sponge-Based

**Figure 13:**
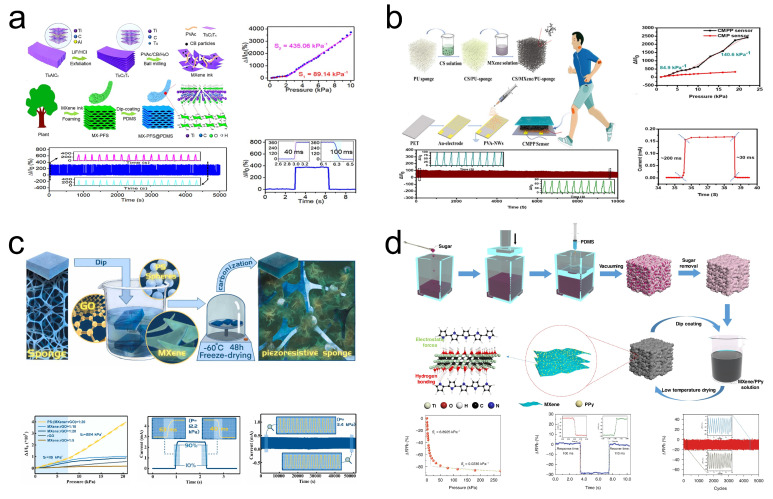
(**a**) Preparation of MX-PFS and its performance in pressure sensing. Reprinted with permission from Ref. [94]. Copyright 2022, American Chemical Society. (**b**) Preparation of CMPP and its performance in pressure sensing. Reprinted with permission from Ref. [95]. Copyright 2021, American Chemical Society. (**c**) Preparation of MGP and its performance in pressure sensing. Reprinted with permission from Ref. [96]. Copyright 2022, Elsevier. (**d**) Preparation of MPP and its performance in pressure sensing. Reprinted with permission from Ref. [97]. Copyright 2023, Nature.

In addition to being composite of materials mentioned previously for the preparation of pressure sensors, MXene can also be combined with common materials in everyday life. Sponge, a porous material frequently encountered in daily settings, is recognized for its high porosity and significant internal surface area, offering promising applications in the field of pressure sensing [109]. Chen et al. [94] developed a compressible and conductive 3D MXene plant fiber sponge (MX-PFS) (Figure 13a). Tests revealed that the MX-PFS@PDMS sensor based on MX-PFS achieved sensitivity responses of 89.14/kPa and 435.06/kPa within pressure ranges of 0.02–2 kPa and 2–10 kPa, respectively. Moreover, the inherent elasticity of PDMS and the layered porous structure of MX-PFS@PDMS endowed the sensor with excellent response/recovery times (40/100 ms) and maintained stability in electrical response after 5000 cycles of compression loading/unloading tests at 50% strain. Li et al. [95] developed a 3D pressure sensor based on CS/MXene/PU sponge/PVA (CMPP) (Figure 13b). CMPP demonstrated sensitivity responses of 84.9/kPa and 140.6/kPa within pressure ranges of 0.05–5 kPa and 6–22 kPa, respectively, and exhibited good performance in response time (200 ms) and recovery time (30 ms). Thanks to the electrostatic interaction between positively charged chitosan and negatively charged MXene, CMPP could be stirred in deionized water and ultrasonically cleaned for 5 h, yet still function stably for 2000 cycles, showing excellent hydrophobicity.

Furthermore, the excellent elasticity of the sponge offers significant potential for it to serve as a supportive material for other pressure sensor materials. Li et al. [96] devised an MXene/rGO/PS (MGP) sponge pressure sensor by embedding MXene/rGO aerogel clusters filled with PS spheres into a PU sponge. The sponge framework encapsulating the aerogel, along with the embedded aerogel clusters, generates additional conductive pathways during compression. The evaluation revealed that the sensitivity of the MCG sponge pressure sensor was 115/kPa in the region below 7.58 kPa and increased to 224/kPa in the 7.58–20.65 kPa range. Moreover, the excellent elasticity and porous characteristics of the PU sponge, combined with MXene’s outstanding mechanical properties, endowed the MCG sponge pressure sensor with remarkable stability (maintaining sensing performance stability after 15,000 cycles of reciprocal pressure testing at 3.4 kPa) and rapid response/recovery times (63/40 ms). In another study, Xia et al. [97] fabricated MXene/PPy@PDMS (MPP) sponges by coating PDMS sponge frameworks obtained via sacrificial sugar templating with MXene/PPy solution synthesized through in situ polymerization. The evaluation demonstrated that the MPP sponge pressure sensor achieved high sensitivity (6.8925/kPa), short response/recovery times (100/110 ms), excellent stability (5000 cycles), and a low detection limit (below 0.43 Pa) within the pressure range of 0–15 kPa. Notably, with the assistance of CNN deep learning algorithms, the MPP pressure sensor could accurately identify human movements, showcasing significant potential in the field of intelligent wearable sensing.

## 4. MXene Composite-Based Gas Sensors

Gases are integral to both everyday life and various production sectors, necessitating the precise detection of chemical substances in the air to safeguard public health and ensure the efficiency of production activities in the food, medical, environmental, and industrial domains. With the advancement of society’s productive forces, the demand for room-temperature, flexible, intelligent gas detection devices, suitable for both indoor and outdoor applications, has significantly increased [110]. Traditional gas sensors, mainly composed of semiconductor materials, have been extensively used in gas detection but are limited by their high operational temperatures, which affect their sensitivity and response times at room temperature [111,112,113,114]. Consequently, 2D nanostructured materials have garnered interest among researchers aiming to achieve accurate and efficient gas detection at room temperature. Two-dimensional nanostructures are considered ideal for gas sensors due to their high surface area, multifunctional surface chemistry, and sensitivity at room temperature [115,116]. Among the family of 2D nanostructured materials, MXenes stand out for their excellent mechanical properties, rich surface functional groups, and good conductivity [82], making them widely applied in the research of flexible gas sensors [32,117,118]. An ideal gas sensor should exhibit high sensitivity, a low limit of detection (LOD), and fast response times. This section summarizes the latest developments in flexible gas sensors for detecting both inorganic gases and volatile organic compounds, highlighting significant advancements in recent years. Detailed performance metrics of the room temperature gas sensors can be found in Table 3.

### 4.1. NO_2_

Over the past centuries, human society has made significant progress with the advancement of science and technology. However, this progress has been accompanied by a series of gas pollution issues. Nitrogen dioxide (NO_2_), a reddish-brown gas with an irritating odor and flammable properties, is among these pollutants. Prolonged exposure to excessive NO_2_ can lead to various types of pulmonary inflammation, including chronic bronchitis, emphysema, and asthma, posing a severe threat to human health [131]. Furthermore, acid rain associated with NO_2_ also causes substantial damage to social production activities, such as agriculture. Therefore, developing NO_2_ gas sensors with high sensitivity and LOD is of paramount importance.

**Figure 14:**
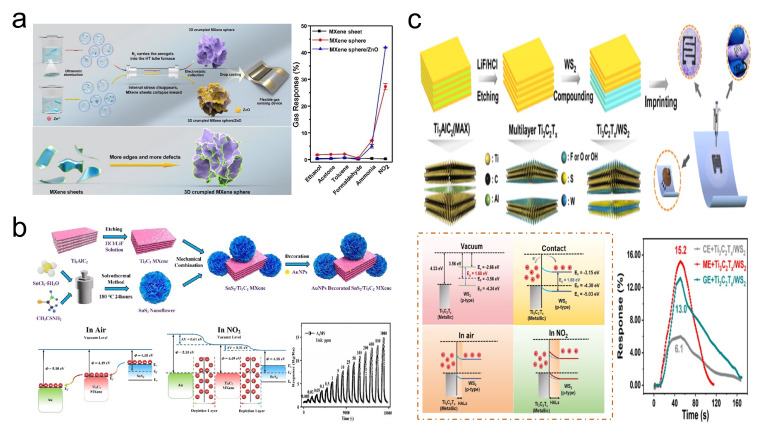
(**a**) Preparation of 3D crumpled spherical MXene/ZnO nanomaterials and its schematic mechanism of crumpled spherical structure and performance in NO_2_ gas sensing. Reprinted with permission from Ref. [119]. Copyright 2021, Elsevier. (**b**) Preparation of MXene@Au/SnS_2_ composite material and its band structure diagram and performance in NO_2_ gas sensing. Reprinted with permission from Ref. [120]. Copyright 2023, Elsevier. (**c**) Preparation of Ti_3_C_2_T_*x*_/WS_2_ thin film composite material and its band structure diagram and performance in NO_2_ gas sensing. Reprinted with permission from Ref. [118]. Copyright 2023, American Chemical Society.

Optimizing the structural design of MXene materials is a viable strategy to enhance the performance of MXene gas sensors. Despite MXenes being considered ideal for gas sensing applications, 2D MXene materials experience significant agglomeration upon drying. This leads to a reduction in their advantageous high specific surface area, consequently diminishing the response of sensors constructed from MXene materials. To address this challenge, the Yang group [119] introduced a flexible gas sensor made from a composite of 3D crumpled spherical MXene Ti_3_C_2_T_*x*_ and ZnO nanomaterials (Figure 14a). Furthermore, the Yang group provided an optimization mechanism for the sensing performance through the 3D crumpled sphere structure. Three-dimensional crumpled MXene spheres not only offer an increased specific surface area but also the ridges formed on the surface of these 3D crumpled MXene spheres by MXene folding disrupt internal chemical bonds and expose more line defects. This consequently provides more gas adsorption sites, thereby enhancing the sensor’s sensing performance. According to the Yang group’s testing, the 3D MXene/ZnO sensor demonstrated a response of 41.93% in a 100 ppm NO_2_ environment, significantly higher than the 27.27% response observed for a sensor made purely of 3D MXene. In another study, Gasso et al. [121] utilized a hydrothermal method to fabricate NO_2_ gas sensors based on SnO_2_/MXene heterostructures capable of ppb-level detection. The excellent ppb-level detection performance of the SnO_2_/MXene heterostructure NO_2_ gas sensor is attributed to the high electronegativity and intrinsic oxygen vacancies of SnO_2_, coupled with the increased reaction sites due to the reduced agglomeration facilitated by the MXene-SnO_2_ heterostructure. Tests showed that this NO_2_ gas sensor exhibited a 231% response to 30 ppb NO_2_, with a response time/recovery time of 146/102 s.

The utilization of MXene composites represents a promising avenue for enhancing the performance of MXene gas sensors. Sulfide materials are often considered for NO_2_ gas sensing applications [132]. Tin disulfide (SnS_2_), a traditional and significant layered transition metal sulfide, is widely applied in NO_2_ detection due to its narrow bandgap (2.18–2.44 eV), high specific surface area, and high surface activity [133,134,135]. Although most previously reported SnS_2_ gas sensors for NO_2_ detection to operate above room temperature [136], fortunately, this issue can be addressed by introducing carbon family materials [137]. Not only does the formation of a heterojunction between Ti_3_C_2_T_*x*_ and SnS_2_ increase the number of electrons flowing towards NO_2_, thereby enhancing the material’s response to NO_2_, but the electron depletion layer formed between Au and Ti_3_C_2_T_*x*_ also provides additional active adsorption sites for NO_2_. Zhang et al. [120] proposed a gas sensor for detecting NO_2_ at room temperature based on a Ti_3_C_2_T_*x*_ MXene@Au/SnS_2_ composite material (Figure 14b). This sensor achieved a response of 5.34 at 1 ppm NO_2_, with an LOD of 5 ppb. Additionally, tungsten disulfide (WS_2_) is considered a highly promising 2D material for NO_2_ detection due to its excellent adsorption properties, abundance of active sites, and exceptional response to redox reactions. Quan et al. [118] designed a flexible paper-based gas sensor using a Ti_3_C_2_T_*x*_/WS_2_ thin film (Figure 14c). Leveraging the heterojunction layers formed between Ti_3_C_2_T_*x*_/WS_2_ and the large contact area provided by the Ti_3_C_2_T_*x*_/WS_2_ heterostructure for gas sensing performance enhancement, the sensor by Quan et al. demonstrated a 15.2% response to 1 ppm NO_2_ and an LOD of 11 ppb.

### 4.2. NH_3_

Ammonia (NH_3_) is a gas of immense importance to humans. NH_3_ and its aerosol product (${\mathrm{NH}}_{4}^{+}$) have been widely used in the chemical industry, including in fertilizers, plastics, and synthetic fibers [138]. Additionally, human metabolic activities and the decomposition of protein-rich foods also produce NH_3_ gas [139,140]. However, NH_3_ is also a pollutant. Prolonged exposure to NH_3_, even at low concentrations, can harm human health, leading to vomiting, headaches, and pulmonary edema, among other illnesses [141]. Therefore, accurately predicting the concentration of NH_3_ in the air is of significant importance for both human health and industrial production. Traditional NH_3_ sensors based on metal oxides suffer from several drawbacks, such as high operating temperatures (>300 °C), poor selectivity, and poor stability in the presence of water. Thus, the development of NH_3_ sensors with high response, LOD, and the capability for room-temperature detection is critically important.

Gas sensors based on organic conductive polymers offer advantages such as tunable electrical properties, environmental stability, flexibility, and the ability to operate at room temperature. However, gas sensors based on pristine conductive polymers typically exhibit lower electrical performance, limiting the application of organic conductive polymer-based gas sensors [7]. Fortunately, this issue can be addressed by combining conductive polymers with 2D materials like MXene, which possess higher conductivity.

**Figure 15:**
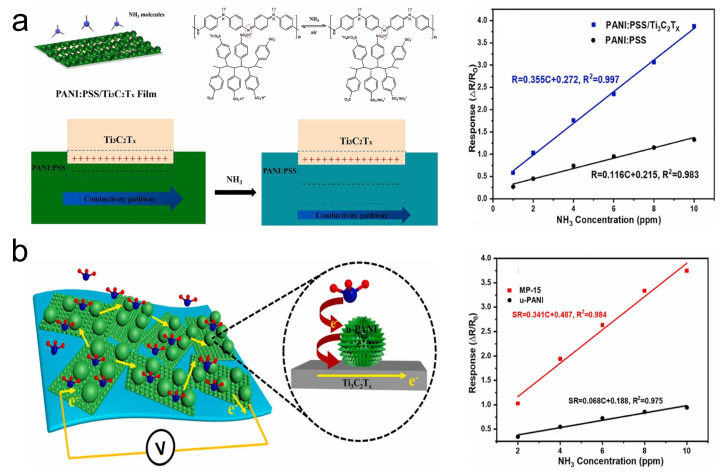
(**a**) Schematic mechanism and performance of Polyaniline (PANI): PSS/Ti_3_C_2_T_*x*_ film in NH_3_ gas sensing. Reprinted with permission from Ref. [122]. Copyright 2022, Elsevier. (**b**) Schematic mechanism and performance of Ti_3_C_2_T_*x*_ MXene nanosheets/urchin-like Polyaniline (PANI) hollow nanosphere composites in NH_3_ gas sensing [123]. Copyright 2022, Elsevier.

Polyaniline (Polyaniline (PANI)) is one of the representative conductive polymers known for its reversible doping/dedoping characteristics, excellent conductivity, and room-temperature sensing capabilities, making it a material with significant potential for room-temperature NH_3_ sensing applications [142,143]. On the one hand, to enhance the sensing performance of MXene/Polyaniline (PANI) gas sensors, introducing Polyaniline (PANI)-related materials with suitable properties is a common strategy. Polyaniline (PANI) doped with Polyaniline (PANI): PSS also exhibits high water dispersion stability and a narrow particle size distribution [144], making it a favorable choice. Wen et al. [122] synthesized Polyaniline (PANI): PSS/Ti_3_C_2_T_*x*_ composites via an in situ polymerization method and introduced the sensing mechanism of this material (Figure 15a). The rich Schottky heterojunctions formed in Polyaniline (PANI): PSS/Ti_3_C_2_T_*x*_, the introduction of a large number of -SO_3_H groups by PSS anionic chains, and the increased protonation level of the Polyaniline (PANI) component, collectively endow the Polyaniline (PANI): PSS/Ti_3_C_2_T_*x*_ composite with excellent sensing performance for NH_3_ detection. According to tests by Wen et al., this material demonstrated a response of 57% to 1 ppm NH_3_, with an LOD of 20 ppb. On the other hand, optimizing sensor sensitivity by introducing biomimetic structures represents another research-worthy direction. Inspired by the structure of sea urchins, Cai et al. [123] employed a templating method and in situ polymerization to prepare a high-performance NH_3_ sensor based on Ti_3_C_2_T_*x*_ MXene nanosheets/urchin-like Polyaniline (PANI) hollow nanosphere composites (MP) (Figure 15b). According to Cai et al.’s testing, this sensor exhibited a response of 3.70 to 10 ppm NH_3_, with an LOD of 30 ppb. In another study, Yang et al. [124] utilized Polyaniline (PANI) and Ti_3_C_2_T_*x*_ nanosheets to fabricate a Polyaniline (PANI)/Ti_3_C_2_T_*x*_ flexible sensor with a 3D mesh-like structure through electrospinning technology. The enhancement in sensing performance of the Polyaniline (PANI)/Ti_3_C_2_T_*x*_ flexible sensor is attributed to the increased degree of Polyaniline (PANI) protonation facilitated by the Schottky junctions formed on the surfaces of Polyaniline (PANI) and Ti_3_C_2_T_*x*_, as well as the fibrous structure. The sensor exhibited a response of 55.90% towards 20 ppm NH_3_. Additionally, under various bending angles (up to a maximum compression of 150°) and bending cycles (up to 3200 cycles), the Polyaniline (PANI)/Ti_3_C_2_T_*x*_ flexible sensor maintained a response to 20 ppm NH_3_ at approximately 55.90%. This suggests its potential for future applications in wearable NH_3_ gas sensors.

### 4.3. Acetone

Acetone is an organic volatile compound widely used in laboratories and industrial production [145]. Research indicates that even trace amounts of prolonged exposure can cause irreversible harm to the human body [146]. Additionally, acetone serves as a significant indicator of human lipid metabolism activities [147]. Given its colorless nature, developing acetone gas sensors for use in daily life holds significant importance.

**Figure 16:**
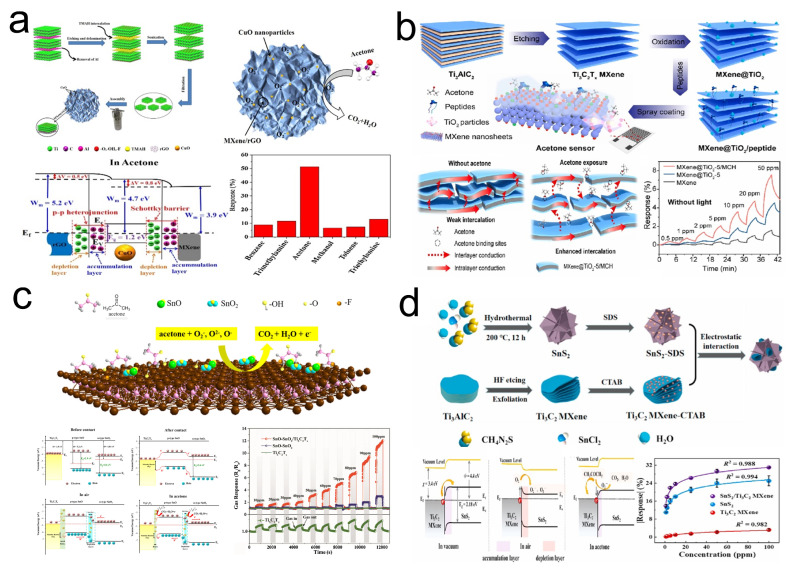
(**a**) Preparation of 3D MXene/rGO/CuO and its schematic mechanism, band structure diagram, and performance in acetone gas sensing. Reprinted with permission from Ref. [125]. Copyright 2021, Elsevier. (**b**) Preparation of Ti_3_C_2_T_*x*_ MXene@TiO_2_-5/MCH and its schematic mechanism and performance in acetone gas sensing. Reprinted with permission from Ref. [126]. Copyright 2023, Elsevier. (**c**) Schematic mechanism of Ti_3_C_2_T_*x*_/SnO-SnO_2_ nanocomposites and its band structure diagram and performance in acetone gas sensing. Reprinted with permission from Ref. [127]. Copyright 2021, Elsevier. (**d**) Preparation of SnS_2_/Ti_3_C_2_ MXene composite materials and its band structure diagram and performance in acetone gas sensing. Reprinted with permission from Ref. [128]. Copyright 2023, Elsevier.

Metal oxides are common materials used in traditional gas sensors [145,148]. However, their operation at temperatures far above room temperature limits their use in daily life. Fortunately, studies have shown that gas sensors using composites of MXene and metal oxides can detect acetone at room temperature [149]. Notably, due to their 2D layered structure, MXene sheets are prone to stacking, which reduces their specific surface area and affects the sensor’s sensitivity. Introducing graphene to construct a 3D hydrogel structure is a viable solution. Liu et al. [125] proposed a 3D hybrid aerogel decorated with CuO nanoparticles (3D MXene/rGO/CuO). Compared to the original MXene/CuO materials, the 3D MXene/rGO/CuO structure offers a large specific surface area, and the Schottky barriers established at the interfaces between CuO nanoparticles and rGO nanosheets provide excellent room-temperature acetone detection performance (Figure 16a). This material showed a response of 52.09% to 100 ppm of acetone. Furthermore, Ti_3_C_2_T_*x*_ MXene materials, rich in Ti sources, allow for the growth of TiO_2_ to be controlled by adjusting the thermal oxidation temperature, duration, and medium. The growth of TiO_2_ facilitates the formation of heterojunctions and atomic defects, enhancing gas sensitivity [150]. Li’s group [126] introduced a composite material based on Ti_3_C_2_T_*x*_, MXene@TiO_2_, and tripeptide Met-Cys-His (MCH)—Ti_3_C_2_T_*x*_ MXene@TiO_2_-5/MCH (Figure 16b). This gas-sensitive material achieved a response of 7.56% to 50 ppm of acetone and an LOD of 0.22 ppm at room temperature. Doping oxides to improve the performance of gas-sensitive materials is also a common approach. Wang et al. [127] successfully synthesized SnO-SnO_2_/Ti_3_C_2_T_*x*_ MXene nanocomposites in situ via a one-step hydrothermal method (Figure 16c). This composite material demonstrated a response value of 12.1 to 100 ppm of acetone, which is four times higher than that of the original SnO-SnO_2_ (3.0) and eleven times higher than that of Ti_3_C_2_T_*x*_ (1.1). Compared to the original SnO-SnO_2_ and Ti_3_C_2_T_*x*_ materials, the Ti_3_C_2_T_*x*_/SnO-SnO_2_ composite material’s larger specific surface area, Schottky barriers, and more extensive oxygen vacancies further enhanced its gas sensitivity.

2D metal/chalcogenides (MCs), such as MoS_2_, InSe, and SnS_2_, are commonly used gas-sensitive materials in gas sensors [67,136,151]. Research indicates that doping semimetal nanomaterials into MCs to form heterostructures is an effective strategy to enhance the gas sensitivity of MCs [152]. Wu et al. [128] assembled SnS_2_/Ti_3_C_2_ MXene composite materials through electrostatic interactions and deposited the aerosol on a flexible substrate to construct a sensing platform for gas detection (Figure 16d). The Schottky barriers formed between Ti_3_C_2_ and SnS_2_, along with the oxygen-containing functional groups on the surface of Ti_3_C_2_, facilitate the adsorption of oxygen-containing substances, which is beneficial for improving gas sensitivity. Furthermore, Ti_3_C_2_ exhibits high electron transport capability, further enhancing gas sensitivity. According to tests by Wu et al., this sensing platform demonstrated a response of 29.8% to 50 ppm of acetone at room temperature, with an LOD of 5.5 ppb.

### 4.4. Ethanol

Ethanol is a common organic solvent in both laboratories and everyday life, often used for the extraction of organic compounds. Due to its volatile nature at room temperature, excessive ethanol vapor in the air can easily lead to explosions when exposed to an open flame. Therefore, developing accurate gas sensors for detecting ethanol is crucial for ensuring the safety of lives and property in specific settings. Studies have shown that sensors made from Ti_3_C_2_T_*x*_ MXene films exhibit the highest response to ethanol compared to other gases [153]. Density Functional Theory simulations and bulk electrical sensitivity measurements indicate that MXene/Ag composite materials demonstrate higher gas sensitivity to ethanol (with adsorption energies of 1.02 eV for MXene and 1.76 eV for MXene/Ag, respectively) compared to pristine MXene films. Based on this finding, Wang’s group [129] proposed an ethanol sensor based on Ti_3_C_2_T_*x*_ MXene/Ag material. This sensor achieved a response of 2.04 to 100 ppm of ethanol, which is 120 times greater than that of the original Ti_3_C_2_T_*x*_ MXene film (1.7%). In another study, Wu et al. [130] fabricated a flexible PP/Ti_3_C_2_T_*x*_/PPy composite gas sensor by drop-coating a Ti_3_C_2_T_*x*_ MXene suspension onto the surface of a disposable mask (primarily made of PP) and conducting pyrrole chemical polymerization. The enhancement in sensing performance of the PP/Ti_3_C_2_T_*x*_/PPy composite material is attributed not only to the influence of its internal Schottky junctions but also to the abundant hydrogen bonding formed between Ti_3_C_2_T_*x*_, PPy, and ethanol molecules, which enlarges the distortion of PPy molecular chains, thereby reducing the electrical conductivity of the PP/Ti_3_C_2_T_*x*_/PPy composite material and improving its responsiveness to ethanol. The sensor exhibited a sensing response, response time, and recovery time of 76.3%, 49 s, and 18 s, respectively, towards 400 ppm ethanol, and it could also detect ethanol concentrations as low as 2.21 ppm. Furthermore, based on this PP/Ti_3_C_2_T_*x*_/PPy composite gas sensor, Wu et al. designed a wireless sensing system with Bluetooth functionality, providing a promising example for applications in alcohol detection such as drunk-driving prevention.

## 5. Conclusions and Outlook

In the preceding sections, we summarized the latest developments in the field of wearable devices, focusing on strain sensors, pressure sensors, gas sensors, and biosensors based on MXene and its composites, through an introduction to some typical examples of sensor performance optimization and biocompatibility. In terms of sensor performance optimization, researchers have primarily focused on optimizing the chemical properties and structural design of MXene materials and their composites, successfully enhancing the sensing capabilities of these sensors in wearable applications. Additionally, researchers are not only dedicated to improving the sensing performance of MXene-based composite materials but also focused on enhancing other properties beyond sensing, such as EMI shielding, thermal management, degradability, and antibacterial properties. Therefore, the introduction of MXene composites can provide significant momentum for the development of MXene-based flexible sensor applications. Although extensive research has been conducted on flexible sensors based on MXene materials, there are still some challenges to be addressed in their application in the wearable domain.

The primary step in the large-scale production and application of MXene-based flexible sensors is the synthesis of MXene itself. Currently, the mainstream synthesis methods for MXene materials can be categorized into two approaches: fluorine-containing synthesis (e.g., HF etching method and LiF/HCl etching method) and fluorine-free synthesis (e.g., alkaline solution etching method and electrochemical etching method). Fluorine-containing synthesis is presently the most rapid and efficient method for obtaining high-quality MXene through quick exfoliation. However, the harmful fluorine-containing waste generated during the synthesis process poses significant concerns for both safety during production and environmental impact. Fluorine-free synthesis, in comparison, offers advantages in terms of safety and environmental impact during the production process compared to fluorine-containing synthesis. Nevertheless, it falls short in efficiency and quality compared to fluorine-containing synthesis in the synthesis of MXene. Therefore, the quest for more efficient, high-quality, safe, and environmentally friendly methods for MXene synthesis is crucial to drive the large-scale production and application of MXene in flexible sensors and other fields. Additionally, for applications in sensors, sensitivity is a crucial metric for evaluating performance. However, the standards and methods used to measure the sensitivity of sensors vary. For instance, in the case of biosensors, Zhang and colleagues developed a sensor for detecting K^+^ with sensitivity measured in “mV/dec”, where “dec” refers to a tenfold change in concentration [154]. Conversely, the Li group used “mg/mL” as the unit for measuring the sensitivity of their sensor for detecting Pb^2+^ [155]. Beyond the inconsistency in sensitivity units, the testing equipment and environments also differ, hindering parallel comparisons of sensor performance across different devices. Moreover, the operational performance of MXene-based flexible sensors under extreme conditions requires improvement. While MXene-based flexible sensors have demonstrated excellent performance under laboratory conditions, their application in real-world scenarios necessitates consideration of complex or even extreme working environments. Sensor performance can be significantly influenced by temperature, humidity, gas concentration, and other factors. For example, MXene tends to oxidize in humid environments, greatly affecting its conductivity and, consequently, its sensing performance. Although there are MXene composite material sensors designed for extreme environments, their lower sensing performance limits practical applications. Therefore, developing MXene-based sensors capable of withstanding various extreme conditions is a critical step in transitioning from laboratory to real-world applications.

## Figures and Tables

**Table 1:** Performance of MXene composite-based strain sensor properties in this review.

Material	GF (Test Range)	Response Time	Recovery Time	Lowest Detection	Ref.
MXene/AgNW	39 (25–40%)	610 ms	620 ms	/	[43]
MXene/AgNW	1085 (80–100%)	340 ms	320 ms	/	[44]
MXene/AgNW	4720 (80–120%)	/	/	0.0645%	[45]
MXene/AgNW	468 (51–68%)	200 ms	/	0.1%	[46]
MXene/CNT/TPU	2911 (250–330%)	80 ms	/	0.1%	[47]
MXene/CNT	9022 (190–210%)	140 ms	160 ms	0.1%	[48]
MXene/multi- walled carbon nanotube (MWCNT)	363 (80–101%)	/	/	/	[49]
MXene/reduced graphene oxide (rGO)	84,326 (160–200%)	70 ms	/	0.05%	[50]
MXene/single-walled carbon nanotubes (SWCNT)	3400 (3–6%)	64 ms	82 ms	/	[51]
MXene/CNF	12.29 (530–800%)	100 ms	140 ms	/	[52]
MXene/CNF/ acrylic acid (AA)/acrylic acid-N- hydrosuccinimide ester (AA-NHS)/ sulfobetaine methacrylate (SMBA) hydrogel	4.98 (1750–2240%)	95 ms	198 ms	1%	[53]
MXene/CNF/Fe_3_O_4_ hydrogel	0.56 (0–181.5%)	/	/	/	[54]
MXene/polyvinyl alcohol (PVA)/ ZnSO_4_ hydrogel	5.82 (100–180%)	/	/	3%	[55]
MXene/polyacrylamide/ sodium alginate hydrogel	2.83 (0–120%)	400 ms	300 ms	/	[56]
MXene/agarose/ polyacrylamide hydrogel	3.38% (@5%)	/	/	/	[57]
MXene/PVA/catechol	2.3 (0–200%)	/	/	/	[58]
MXene/gelatin	2.8 (0–100%)	400 ms	/	/	[59]

**Table 2:** Performance of MXene composite-based pressure sensor properties in this review.

Material	Sensitivity (Test Range)	Response Time	Recovery Time	Lowest Detection	Ref.
MXene/AgNW	474.48/kPa (0–1.25 kPa)	140 ms	30 ms	1 Pa	[86]
MXene/AgNW	770.86/kPa (0–0.75 kPa)	70 ms	81 ms	1 Pa	[87]
MXene/carbon nanofiber	65/kPa (<10 Pa)	46 ms	26 ms	5 Pa	[88]
MXene/MWCNT/TPU	132/kPa (0–5.7 Mpa)	260.8 ms	176 ms	/	[89]
MXene/carboxylated CNT/carboxymethyl chitosan	3.84/kPa (0–12.4 kPa)	62 ms	/	/	[90]
MXene/CNF/CNT	817.3/kPa (0–200 Pa)	74 ms	50 ms	/	[91]
MXene/CNF	649.3/kPa (8.04–20.55kPa)	123 ms	139 ms	/	[92]
MXene/CNF/CS	117.08/kPa (0–1 kPa)	6.3 ms	8.1 ms	3 Pa	[93]
3D MXene plant fiber sponge	435.06/kPa (2–10 kPa)	40 ms	100 ms	20 Pa	[94]
MXene/PU sponge/PVA/chitosan	140.6/kPa (6–22 kPa)	200 ms	30 ms	/	[95]
MXene/rGO/ polystyrene (PS)/PU sponge	224/kPa (7.58–20.65 kPa)	63 ms	40 ms	/	[96]
MXene/polypyrrole (PPy)/ PDMS sponge	6.8925/kPa (0–15 kPa)	100 ms	110 ms	<0.43 Pa	[97]

**Table 3:** Performance of MXene composite-based gas sensor properties in this review (room temperature).

Target Gas	Material	Response (Concentration)	Response Time	Recovery Time	LOD	Ref.
NO_2_	3D MXene sphere/ZnO	41.93% (100 ppm)	34 s	103 s	/	[119]
NO_2_	MXene@Au/SnS_2_	5.34 (1 ppm)	310 s	179 s	0.005 ppm	[120]
NO_2_	MXene/WS_2_	15.2% (1 ppm)	/	70 s	11.0 ppb	[118]
NO_2_	MXene/SnO_2_	231% (30 ppb)	146 s	102 s	/	[121]
NH_3_	MXene/polystyrene sulfonate (PANI: PSS)	57% (1 ppm)	276 s	388 s	20 ppb	[122]
NH_3_	MXene/urchin-like Polyaniline (PANI) hollow nanosphere	3.7 (10 ppm)	275 s	414 s	30 ppb	[123]
NH_3_	PANI/Ti_3_C_2_T_*x*_	55.90% (20 ppm)	/	/	/	[124]
Acetone	3D MXene/rGO/CuO aerogel	52.09% (100 ppm)	6.7 s	7.5 s	/	[125]
Acetone	MXene@TiO_2_-5/MCH	7.56% (50 ppm)	/	/	0.22 ppm	[126]
Acetone	MXene/SnO-SnO_2_	12.1 (100 ppm)	18 s	9 s	/	[127]
Acetone	MXene/SnS_2_	23.6 (10 ppm)	92 s	132 s	5.5 ppb	[128]
Ethanol	MXene/Ag	2.04 (100 ppm)	/	/	/	[129]
Ethanol	Ti_3_C_2_T_*x*_/PPy/polypropylene (PP)	76.3% (400 ppm)	49 s	18 s	2.21 ppm	[130]

## Data Availability

No new data were created or analyzed in this study.
